# Immune response to allogeneic equine mesenchymal stromal cells

**DOI:** 10.1186/s13287-021-02624-y

**Published:** 2021-11-12

**Authors:** J. Lacy Kamm, Christopher B. Riley, Natalie A. Parlane, Erica K. Gee, C. Wayne McIlwraith

**Affiliations:** 1 School of Veterinary Science, Massey University, Tennent Drive, Palmerston North, 4442 New Zealand; 2 AgResearch, Hopkirk Research Institute, Massey University, Palmerston North, 4474 New Zealand; 3 C. Wayne McIlwraith Translational Medicine Institute and the Orthopaedic Research Center, Colorado State University, 1678 Campus Delivery, Fort Collins, CO 80523-1678 USA

**Keywords:** Allogeneic, Equine, Immune, Lymphocyte

## Abstract

**Background:**

Mesenchymal stromal cells (MSCs) are believed to be hypoimmunogeneic with potential use for allogeneic administration.

**Methods:**

Bone marrow was harvested from Connemara (*n* = 1), Standardbred (*n* = 6), and Thoroughbred (*n* = 3) horses. MSCs were grouped by their level of expression of major histocompatibility factor II (MHC II). MSCs were then sub-grouped by those MSCs derived from universal blood donor horses. MSCs were isolated and cultured using media containing fetal bovine serum until adequate numbers were acquired. The MSCs were cultured in xenogen-free media for 48 h prior to use and during all assays. Autologous and allogeneic MSCs were then directly co-cultured with responder leukocytes from the Connemara horse in varying concentrations of MSCs to leukocytes (1:1, 1:10, and 1:100). MSCs were also cultured with complement present and heat-inactivated complement to determine whether complement alone would decrease MSC viability. MSCs underwent haplotyping of their equine leukocyte antigen (ELA) to determine whether the MHC factors were matched or mismatched between the donor MSCs and the responder leukocytes.

**Results:**

All allogeneic MSCs were found to be ELA mismatched with the responder leukocytes. MHC II-low and universal blood donor MSCs caused no peripheral blood mononuclear cell (PBMC) proliferation, no increase in B cells, and no activation of CD8 lymphocytes. Universal blood donor MSCs stimulated a significant increase in the number of T regulatory cells. Neutrophil interaction with MSCs showed that universal blood donor and MHC II-high allogeneic MSCs at the 6 h time point in co-culture caused greater neutrophil activation than the other co-culture groups. Complement-mediated cytotoxicity did not consistently cause MSC death in cultures with active complement as compared to those with inactivated complement. Gene expression assays revealed that the universal blood donor group and the MHC II-low MSCs were more metabolically active both in the anabolic and catabolic gene categories when cultured with allogeneic lymphocytes as compared to the other co-cultures. These upregulated genes included CD59, FGF-2, HGF, IDO, IL-10, IL-RA, IL-2, SOX2, TGF-β1, ADAMSTS-4, ADAMSTS-5, CCL2, CXCLB/IL-8, IFNγ, IL-1β, and TNFα.

**Conclusions:**

MHC II-low MSCs are the most appropriate type of allogeneic MSC to prevent activation of the innate and cell-mediated component of the adaptive immune systems and have increased gene expression as compared to other allogeneic MSCs.

**Supplementary Information:**

The online version contains supplementary material available at 10.1186/s13287-021-02624-y.

## Introduction

The interaction of the immune system with foreign antigens initiates inflammation and allorecognition. When the foreign source of antigenic stimulation is a therapy such as allogeneic mesenchymal stem cells (MSCs), the immune reaction can be detrimental to the survival of the donor cells and, consequently, may impair the intended health benefits for the recipient. MSCs are commonly believed to have innate immunosuppressive properties [16, 17, 21, 22, 26, 27, 65]. Human studies have repeatedly shown that MSCs have immunosuppressive effects via T regulatory (Treg) and B regulatory cell upregulation leading to decreased activation of T lymphocytes and B cells, respectively [13, 63].

Immunosuppression within the recipient site by MSCs is necessary as allogeneic MSCs may be rejected due to their expression of foreign surface antigens. The presence of major histocompatibility class I and II (MHC I and II) surface antigens on equine MSCs (and their specific equine leukocyte antigen (ELA) haplotype) facilitates immune recognition by lymphocytes [2, 48]. Equine bone marrow-derived MSCs express MHC I and variably express MHC II [35, 57]. Mismatched ELA haplotype donor MSCs have been shown to induce greater lymphocyte activation in vitro as compared to matched donor MSCs [57]. When given as a repeat treatment mismatched MSCs may activate an alloantibody response which can target the MSCs for destruction prior to exerting their therapeutic effects [7, 8, 27, 48, 54].

In order to find an MSC that would defer immune recognition, we studied various groups of horses with unique MSC types. In a previous study, we found two groups of horses: one with high levels of MHC II expression on the surface of their MSCs and one with low levels of MHC II expression [35]. Our previous study contained a subset of horses with low expressing MHC II MSCs who were also known to be universal blood donors (Aa, Ca, and Qa erythrocyte antigen negative) [50]. None of the universal donor horses had MSCs with high levels of MHC II expression [35]. For this reason, we sought to determine whether there was a link between being a universal blood donor and having MSCs with low antigen expression which may make them more immune privileged than a non-blood donor.

The broad aim of this study was to determine the behavior and effect of MSC interaction with ELA mismatched responder leukocytes in an unactivated environment. The use of unactivated leukocytes would best demonstrate the degree of immune activation of leukocytes when they come into contact with allogeneic MSCs. We hypothesize that there will be significant differences in the interactions between our different MSC groups (MHC II-low expressing MSCs, MHC II-high expressing MSCs, and universal blood donor MSCs) and the responder leukocytes.

## Methods

### Animals, blood typing, and sample groups

Equine bone marrow was harvested from the sternebrae of Standardbred (*n* = 18) and Thoroughbred (*n* = 18) horses, and a Connemara (*n* = 1) pony following ethics approval by the Massey University Animal Ethics Committee (MUAEC Protocol 15/13) as described in [35]. All horses had no previous history of foreign cell administration including blood transfusion nor allogeneic MSC therapy. The horses were either owned independently or by Massey University, and informed consent for their use was granted by all parties.

All horses were blood typed for Aa, Ca, and Qa antigens. Five mL of blood was collected in anticoagulant tubes (ACD Tube, BD Vacutainer®, San Jose, CA, USA) for blood typing at the Equine Parentage and Animal Services Centre at Massey University. A horse was considered a universal blood donor if it was negative for Aa, Ca, and Qa antigens [29, 59].

Only 10 horses of the original 37 that best fit the following criteria were utilized in further assays. MSCs were selected from the Standardbred and Thoroughbred groups according to their MHC class II expression and blood type. The MHC II expression was determined and described in a previous study [35] Three horses with the lowest MHC class II expression that were not universal blood types were chosen to create an ‘MHC class II-low’ group. These MSCs were from Standardbreds (age 2–9 years). Three horses with the highest MHC class II expression that were not universal blood-donor types were chosen to create an ‘MHC class II-high’ group. These MSCs were from Thoroughbreds (age 4–6 years). Three samples of MSCs were randomly selected from the universal blood donor horses, and all were Standardbreds (age 12–21 years). MSCs from one Connemara pony (age 21 years) were used. This horse also had peripheral blood taken for mononuclear, neutrophil, and serum isolation as this breed is likely to have a different ELA haplotype than Thoroughbreds [53]. All horses’ MSCs were tested in triplicate in each of the assays. Control assays (MSCs or immune cells cultured alone) were also performed in triplicate.

### MSC isolation and culture

MSCs were isolated and cultured from bone marrow as described in [35]. Passage 3 MSCs from the 10 horses chosen as samples (see ‘sample groups’ above) were plated in 48 or 96 well-plates (Greiner Bio-One, Austria) (dependent on the assay) with MSC proliferation media containing alpha modification of Eagle’s medium with 10% equine serum (Horse serum, Thermo Fisher®), 1% penicillin/streptomycin/amphotericin B and 2.5% 1 M HEPES buffer. These MSCs were cultured for 48 h prior to fresh media with leukocytes being added (see below). The MSCs were grown without fetal bovine serum (FBS) to minimize any immune reaction to xeno-contaminants as has previously been seen [34].

The MSCs that we used in our studies had been confirmed as being a pure population of MSCs via marker expression analysis and trilineage testing [35].

### MSC haplotyping using microsatellite analysis

Haplotype analysis was performed as described previously [31]. DNA was isolated from each MSC donor using DNA isolation kits (DNeasy Blood and Tissue, Qiagen, Germantown, MD, USA).

6FAM or NED fluorescently labeled PCR primers for 12 horse intra-MHC microsatellite markers were amplified in six PCR reactions and then pooled into four groups for fragment analysis. PCR products were combined with GeneScan Liz-500 size standard and electrophoresed on an ABI3700 instrument at the Cornell BioResource Center.

Fragment lengths were analyzed using GeneMarker v3.0.1 software (Softgenetics, State College, PA, USA) and exported into Excel for phasing.

### Isolation of peripheral blood mononuclear cells

Following ethics approval by the Massey University Animal Ethics Committee (MUAEC Protocol 18/06), blood was aspirated aseptically from the left jugular vein of one Connemara pony. This was the same pony used for MSC isolation. Blood was placed in heparinized blood tubes (BD Vacutainer®, California, USA) for lymphocyte collection and subsequently processed using Lymphoprep™ (Density 1.077 g/mL, Alere Technologies AS, Norway). Briefly, blood was diluted 1:1 with phosphate-buffered saline (PBS) (Gibco™, Thermo Fisher®). Fifteen ml of Lymphoprep™ was placed in a centrifuge tube, and 30 ml of diluted blood was placed on top of the Lymphoprep™. The tube was centrifuged for 25 min at 1125×*g* at low acceleration and without braking, thereby forming a density gradient. The lymphocyte-rich layer at the interface of the serum and the gradient agent was recovered and the neutrophil-rich pellet then used.

The peripheral blood mononuclear cell (PBMC)-rich layer was washed with PBS. The PBMCs were then diluted in PBMC media composed of RPMI 1610 media (Gibco™, Thermo Fisher®) with 10% autologous equine serum, penicillin–streptomycin (100 µg/ml) (Sigma-Aldrich®, St Louis, MO, USA), and 2-mercaptoethanol (0.1 mM) (Gibco™, Thermo Fisher®).

### Neutrophil isolation

A density gradient using fresh blood from a Connemara pony was performed in the presence of Lymphoprep™ (as described previously). The pellet from the density gradient was used to isolate neutrophils. Thirty-five mL of sterile water was added to the pellet. The centrifuge tube was inverted twice for mixing. Five mL of concentrated PBS (10X) (Gibco™, Thermo Fisher®) was then added and the tube centrifuged for 10 min at 1000* g*. The supernatant was discarded, and the neutrophil-rich pellet was washed in PBS. The neutrophils were cultured in media with alpha modification of Eagle’s medium with 10% autologous equine serum, 1% penicillin/streptomycin/amphotericin B, and 2.5% 1 M HEPES buffer.

### Serum collection

Blood from the Connemara pony was also collected into clot activating tubes (CAT BD Vacutainer®, San Jose, CA, USA). The tubes were incubated at 37 °C for 1 h prior to centrifugation at 3220* g* for 15 min [9]. The serum was harvested and used in co-culture media within 90 min of harvest (active serum). Twenty ml of the serum was inactivated by heating to 56 °C for 30 min (inactive serum) and used only in the complement assay.

### MSC and PBMC co-culture

After the MSCs were incubated for 48 h in media containing equine serum, the media were removed, and PBMCs in PBMC media were added to the MSC wells. PBMCs were added in three different ratios of MSCs to PBMCs: 1:1, 1:10, and 1:100. These ratios are based on published values typical for an equine joint during its normal cycle of reaction to an intra-articular MSC injection [4, 18]. PBMCs without MSCs ± 2.5 µg/ml of pokeweed mitogen as an activation agent (PWM, Sigma-Aldrich, Missouri, USA) served as controls.

PBMCs and MSCs were co-cultured at 37 °C with 5%CO_2_ for 3 or 5 days prior to analysis.

### Tritiated thymidine incorporation assay to assess lymphocyte proliferation

PBMCs and MSCs were co-cultured in triplicate for 3 or 5 days prior to the addition of tritiated thymidine in order to determine whether there was lymphocyte proliferation subsequent to MSC co-culture. Tritiated thymidine assays were performed in a 96 well plate (Greiner Bio-One, Monroe, NC, USA). One µCi of [methyl-^3^H]-Thymidine (PerkinElmer, MA, USA) was added per well, and cells were incubated for a further 18 h. Cells were harvested onto glass fiber mats (Tomtec Harvester, Connecticut, USA) and cell-incorporated radioactivity measured using a scintillation counter (Wallac TriLux MicroBeta 1450, Finland) and reported as counts per minute (cpm).

### Flow cytometry on PBMCs and MSCs

Flow cytometry was performed to assess changes in lymphocyte sub-populations and MSC antigen expression after co-culture. PBMCs and MSCs were tested just prior to co-culture (Day 0) and on Days 3 and 5 of co-culture. Fixable viability dye (Efluor 780™, eBioscience™, Thermo Fisher) was used to assess cell viability in flow cytometry assays [35]. Samples were measured using a flow cytometer (BD FACSVerse™, BD Biosciences, San Jose, CA, USA). All events in the sample were recorded for the leukocyte population and for the MSC population separately. The MSC and leukocyte populations were characterized using the gating hierarchy as shown in Additional file 1 using flow cytometry analysis software (FlowJo LLC, Oregon, USA).

For lymphocyte analysis, antibodies against extracellular CD4 (CVS4, US Biological, Salem, MA, USA) [28], CD8 (CVS8, BioRad, Hercules, CA, USA) [55], CD21 (CA2.1D6, AbCam, Cambridge, UK) [5], and CD25 (RND Systems, Minneapolis, MN, USA) [28] were used in accordance with previous publications. Following permeabilization (FoxP3 Transcription Factor Staining Buffer, eBioscience, San Diego, CA, USA), an intracellular antibody for FOXP3 (FJK-16 s, eBioscience, San Diego, CA, USA) [28] was then used (Additional file 1).

MSCs were stained for MHC I (CVS22, BioRad, Hercules, CA, USA) and MHC II (CVS20, BioRad, Hercules, CA, USA) using antibodies used in previous publications [12, 35]. Dilution and conjugation for all antibodies are shown in Additional file 1.

### MSC and neutrophil co-culture

Neutrophils and MSCs were co-cultured to determine the degree of neutrophil activation subsequent to their interaction. Following 48-h incubation to allow for MSCs adherence to the plate with media containing equine serum, the media were removed, and fresh neutrophils (less than 2 h post-blood draw) in neutrophil media (described above) were added to the MSC wells. Neutrophils were added in the same ratios described for PBMCs. Neutrophils alone ± 2.5 µM activation agent phorbol myristate acetate (PMA; Sigma-Aldrich, St Louis, Missouri, USA) were cultured to serve as controls.

Neutrophils and MSCs were co-cultured at 37 °C with 5%CO_2_ for 6 h or 12 h prior to analysis with flow cytometry.

### Flow cytometry on neutrophils

Neutrophil activation was assessed after co-culture with autologous or allogeneic MSCs. After 6 or 12 h of co-culture, 123-dihydrorhodamine (0.25 µg/sample) was added to each well and incubated for 20 min in the dark at 37 °C. The wells were the placed on ice for 10 min. The cells were then stained with viability dye (Efluor 780™). Ten thousand events in a large cell gate (including MSCs and neutrophils) were recorded and fluorescence used for statistical analysis.

### MSC and complement culture

Complement and the MSC samples were incubated together to determine whether the complement had a cytotoxic effect on the autologous or allogeneic MSCs. Following 48-h incubation to allow for MSCs adherence to the plate with media containing inactivated equine serum (Horse serum, Thermo Fisher), the media were removed and MSC proliferation media containing 30% active or inactivated serum was added. After 1 h, MSCs were stained as described for flow cytometry.

### Flow cytometry on MSCs cultured with complement

Flow cytometry was performed to assess changes in MSC viability after culture with complement. MSCs were tested after 1 h of culture with active or inactivated complement. Fixable viability dye (Efluor 780™, eBioscences™, Thermo Fisher) was used to assess cell viability in flow cytometry assays. Samples were measured using a BD FACSVerse™ (San Jose, CA, USA). Ten thousand events in a leukocyte gate were recorded.

### Gene expression assay

Transcriptional analysis was performed on separated PBMCs and MSCs after 0, 3, or 5 days of co-culture using the nCounter Analysis System (NanoString, Seattle, WA, USA). Anabolic genes assessed were: transforming growth factor (TGF)-β1 protein, fibroblast growth factor (FGF), interleukin 1 receptor antagonist (IL-1RA), indoleamine-pyrrole 2,3-dioxygenase (IDO1), CD59, hepatocyte growth factor (HGF), IL-10, IL-2, vascular endothelial growth factor 2 (VEGF2) and SOX2. Catabolic genes assessed were: tumor necrosis factor α (TNF-α), IL-1β, aggrecanases (ADAMSTS-4, ADAMSTS-5), matrix metalloproteinase (MMP)-13, chemokine ligand 2 (CCL2), C-X-C motif chemokine ligand 8 (CXCL8/IL-8), interferon γ (IFNγ), cyclooxygenase-2 (PTGS-2/ COX-2), and IL6. Two sets of gene-specific probes (along with a reporter probe and a capture probe) were designed by NanoString, and their accession numbers are listed in Additional file 1. Total RNA (85 ± 59 ng per sample) was hybridized using nCounter PlexSet-24 Reagent Pack according to the PlexSet™ Reagents User Manual. After hybridization, samples were vertically pooled and were placed on the automated nCounter Prep Station (NanoString) for purification and were immobilized in the cartridge. This cartridge was then transferred to the nCounter Digital Analyzer for data collection. Data analysis was performed with nSolver™ 4.0 Analysis Software according to user manual. All samples passed the quality control. Positive control normalization was carried out by using the geometric mean of the highest three positive counts. Reference gene normalization was calculated using the geometric mean of counts for the three reference genes GUSB, PPIA, TBP, YWHAZ [52].

### Statistics

Summative and comparative statistical analyses were performed using statistical software (Statistica 11, Statsoft, Tulsa, OK, USA or R version 3.4.3, R Core Development Team). PBMC population analysis, MSC markers, PBMC proliferation, neutrophil activation, MSC survival, and gene expression data were not normally distributed as determined by Shapiro–Wilk’s testing. Data transformation did not produce normally distributed data. MHC I and MHC II marker expression MSCs for the autologous sample and the universal blood donor, MHCII high and MHC II low groups were compared for each of the time points and at each of the three different ratios of MSCs to PBMCs (1:1, 1:10, and 1:100) by Kruskal–Wallis ANOVA by ranks. If a significant difference was identified, post hoc comparisons were then made using the Benjamini–Hochberg method. Significance was identified at *p* < 0.05.

## Results

### MSC haplotyping

The Connemara pony was of a different ELA haplotype than all of the other horses utilized in this study (Supplemental information). Therefore, all allogeneic co-cultures were ELA mismatched.

### MSC and PBMC co-culture

#### MHC I expression was consistently high on all MSC samples while MHC II expression varied

**Fig. 1:**
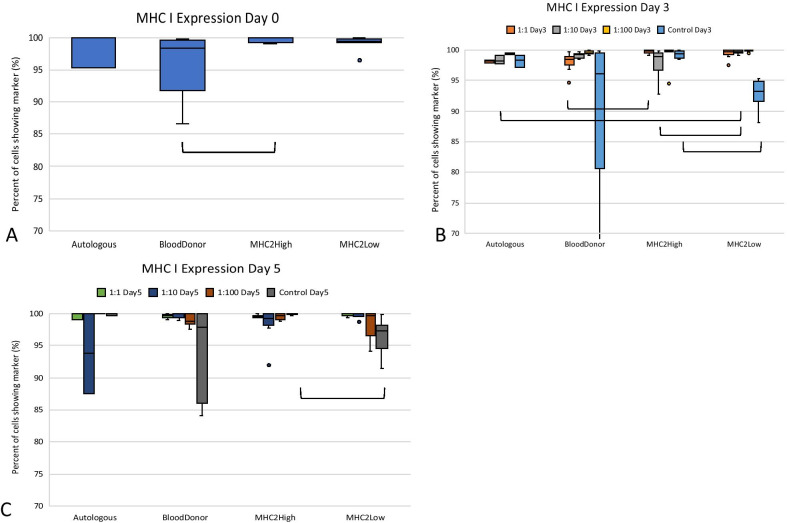
Analysis of MHC I expression on MSCs co-cultured with PBMCs. MHC I expression on MSCs **a** is shown prior to co-culture, **b** after 3 days in co-culture, and **c** at 5 days of co-culture. MHC I expression increased on MSCs in co-culture as compared to their control (non-co-cultured) value (**b**, **c**). Autologous MSCs (*n* = 1); blood donor, universal blood donor MSCs (*n* = 3); MHC II high, MHC II-high MSCs (*n* = 3), MHC II low, MHC II-low MSCs (*n* = 3)

The median value of MHC I expression was greater than 90% for each of the sample groups at Days 0, 3, and 5 of culture (Fig. 1a–c). There were significant differences between the groups as shown in Fig. 1a–c, but due to all the MSCs expressing a high level of MHC I, this will not be discussed further (Table 1).

**Table 1:** MHC I expression on MSCs prior to and during co-culture with PBMCs

	Day 0	Day 3				Day 5			
	Control	1:1	1:10	1:100	Control	1:1	1:10	1:100	Control
Autologous	100 (2.3)^ab^	98.1 (0.2)^a^	98.2 (0.7)^a^	99.4 (0.2)^a^	98.3 (1.0)^a,b^	100 (0.5)^a^	93.8 (6.3)^a^	100 (0)^a^	99.9 (0.2)^a,b^
Blood donor	98.3 (6.1)^a^	98.5 (0.5)^a^	99.2 (0.5)^a,b^	99.5 (0.5)^a^	96.1 (18.2)^a,b^	99.6 (0.3)^a^	100 (0.4)^a^	98.8 (1.4)^a^	97.9 (12.0)^a,b^
MHC II-high	100 (0.6)^b^	99.8 (0.2)^a^	98.9 (2.4)^a^	99.8 (0.2)^a^	99.4 (1.1)^a^	99.5 (0.4)^a^	99.2 (1.4)^a^	99.7 (0.8)^a^	100 (0.2)^a^
MHC II-low	99.5 (0.6)^ab^	99.7 (0.5)^a^	99.7 (0.5)^b^	99.8 (0.1)^a^	93.2 (3.0)^b^	99.9 (0.2)^a^	100 (0.5)^a^	99.7 (2.7)^a^	97.2 (1.6)^b^

Median percent of MSCs expressing MHC I and the interquartile range (IQR) are shown for control cultures (no PBMCs) and for co-cultures with the ratio of MSC/PBMC. Values within each column which have different letters are significantly different (*p* < 0.05). Autologous, autologous MSC co-culture (*n* = 1); blood donor, universal blood donor MSC co-culture (*n* = 3); MHC II high, MHC II-high MSC co-culture (*n* = 3), MHC II low, MHC II-low MSC co-culture (*n* = 3)

**Fig. 2:**
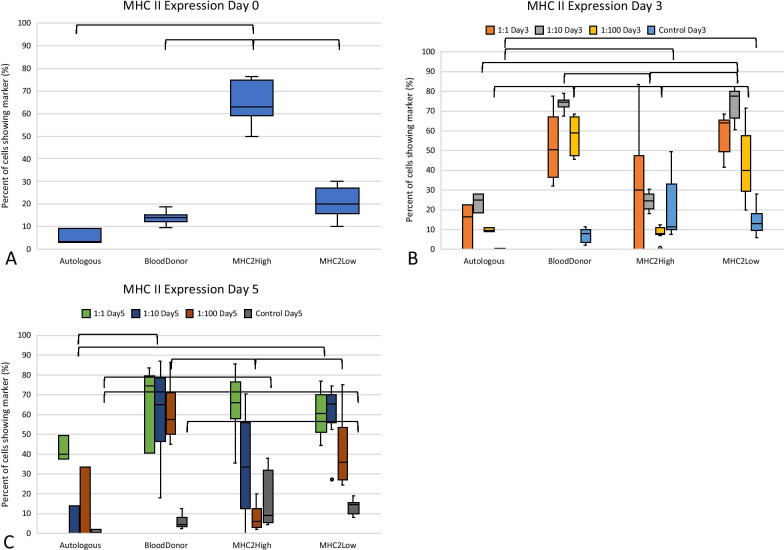
Analysis of MHC II expression on MSCs co-cultured with PBMCs. MHC II expression on MSCs is shown prior to co-culture (**a**), after 3 days in co-culture (**b**), and at 5 days of co-culture (**c**). Increased levels of MHC II expression were seen on MSCs when in co-culture and more specifically when MSCs and lymphocytes were co-cultured at low ratios

MHC II expression was variable at time 0 with the MHC II-high group expressing a significantly greater amount of MHC II antigen (*p* < 0.05) on their surface as compared to the other MSC groups (Fig. 2, Table 2). In co-culture with PBMCs at Days 3 and 5, MSC MHC II expression increased greatly for the universal blood donor and MHC II-low MSC co-cultures. MHC II expression was significantly higher on the blood donor MSCs as compared to the autologous and MHC II-high groups when co-cultured with PBMCs (*p* < 0.05 for both comparisons across all ratios) (Fig. 2, Table 2). The MHC II-low group had significantly greater MHC II expression in co-culture as compared to the autologous samples (*p* < 0.02 for both Day 3 and Day 5) and the MHC II-high group at Day 3 (*p* < 0.001). Additionally, for both the blood donor and the MHC II-low co-culture groups, MHC II expression was significantly higher when the MSCs were co-cultured with PBMCs as compared to their control (MSCs cultured alone) values at Days 3 and 5 (*p* < 0.001) (Fig. 2, Table 2). MHC II expression was not significantly different between the control and co-culture MSCs for the MHC II-high and autologous samples.

**Table 2:** MHC II expression on MSCs prior to and during co-culture with PBMCs

	Day O	Day 3				Day 5			
	Control	1:1	1:10	1:100	Control	1:1	1:10	1:100	Control
Autologous	3.6 (3.2)^a^	16.8 (11.3)^a^	25.0 (4.8)^a,b^	9.7 (1.1)^a,c^	0.42 (0.28)^a^	40.0 (6.1)^a^	0 (7.2)^a^	0 (16.6)^a,b^	0.7 (0.8)^a^
Blood donor	14.2 (2.8)^a^	50.7 (28.5)^a^	74.4 (2.2)^b,c^	59.1 (18.0)^b^	8.0 (5.2)^a,b^	74.2 (37.4)^a^	65.1 (27.9)^b^	57.3 (10.2)^a^	4.6 (3.4)^a,b^
MHC II-HIGH	62.7 (0.6)^b^	30.1 (42.1)^a^	24.3 (7.1)^a^	8.3 (2.9)^a^	11.4 (10.8)^b^	66.1 (14.0)^a^	33.3 (24.5)^a,b^	6.0 (7.4)^b^	9.0 (21.5)^b,c^
MHC II-LOW	20.2 (7.2)^a^	63.7 (11.3)^a^	77.2 (8.7)^c^	40.0 (19.6)^b,c^	13.0 (7.9)^b^	60.7 (13.6)^a^	65.6 (8.0)^b^	36.2 (21.3)^a^	14.5 (4.8)^c^

Median percent of MSCs expressing MHC II and the IQR is shown for control cultures (no PBMCs) and for co-cultures with the ratio of MSC/PBMC. Values within each column which have different letters are significantly different (*p* < 0.05). Autologous, autologous MSC co-culture (*n* = 1); blood donor, universal blood donor MSC co-culture (*n* = 3); MHC II high, MHC II-high MSC co-culture (*n* = 3), MHC II low, MHC II-low MSC co-culture (*n* = 3)

#### Lymphocyte activation was greater in the presence of MHC class II-high MSCs and lower in MHC class II-low MSCs as compared to autologous MSC co-cultures

**Fig. 3:**
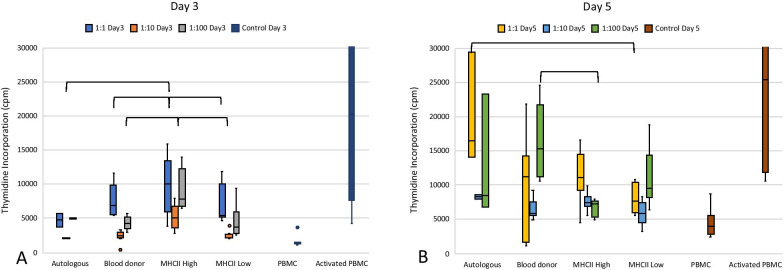
PBMC proliferation in co-culture with MSCs at Day 3 (**a**) and Day 5 (**b**). MHC II-high MSCs caused significantly greater PBMC proliferation on Day 3 than the autologous group. Autologous MSCs caused significantly greater PBMC proliferation than the MHC II-low co-cultures at Day 5. Significant differences between the co-culture groups only are shown with brackets

When MSCs and PMBCs were co-cultured in the presence of tritiated thymidine to assess the level of lymphocyte proliferation, significant differences were observed between MSC groups (Fig. 3, Table 3). At Day 3 of co-culture, autologous MSCs had less tritiated thymidine incorporation linked to less PBMC proliferation than the MHC II-high co-culture when all ratios were combined (*p* = 0.028) and at ratio 1:10 (*p* = 0.016).

**Table 3:** Lymphocyte activation during co-culture with MSCs

	Day 3				Day 5			
	1:1	1:10	1:100	Control	1:1	1:10	1:100	Control
Autologous	4795 (986)^a^	2092 (109)^a^	4973 (86)^a,b^	–	16,435 (7688)^a,#^	8349 (272)^a,#^	8473 (8292)^a,b^	–
Blood donor	6882 (3570)^a,#^	2449 (494)^a^	4319 (1162)^a^	–	11,156 (12,090)^a,b^	5818 (1572)^a^	15,215 (7230)^a,#^	–
MHC II-high	9986 (6166^)a,#^	4996 (2114)^b^	7805 (5145)^b,#^	–	11,135 (3348)^a,b^	7416 (1110)^a,#^	7241 (2015)^b^	–
MHC II-low	5380 (4573)^a^	2202 (317)^a^	3800 (2815)^a^	–	7686 (3961)^b^	5787 (1819)^a^	9493 (5314)^a,b^	–
PBMC	–	–	–	1251 (226)	–	–	–	4048 (2513)
Activated PBMC	–	–	–	20,237 (27,350)	–	–	–	25,342 (29,759)

Median percent of lymphocytes activated and IQR is shown for co-cultures with the ratio of MSC/PBMC and for control cultures with PBMCs cultured alone with or without activating agent. Values within each column which have different letters are significantly different (*p* < 0.05). Values that are significantly greater than the PBMC alone are shown with a #. Autologous, autologous MSC co-culture (*n* = 1); blood donor, universal blood donor MSC co-culture (*n* = 3); MHC II high, MHC II-high MSC co-culture (*n* = 3); MHC II low, MHC II-low MSC co-culture (*n* = 3); PBMCs, PBMCs alone; activated PBMCs, PBMCs activated with 2.5 µg/ml of PWM

At Day 5 of co-culture, MHC class II-low MSCs were associated with significantly less PBMC proliferation than the autologous MSCs across all ratios (*p* = 0.029) and was significantly less at ratio 1:1 (*p* = 0.041) (Table 3). MHC class II-low MSCs had an activation level similar to PBMCs alone and was not associated with a significantly greater amount of proliferation at any ratio at Day 3 nor Day 5. The other two allogeneic groups and the autologous MSCs had significantly greater activation as compared to PBMCs alone at least one time point/ratio combination.

### CD4 lymphocyte counts decreased over time in co-culture

**Fig. 4:**
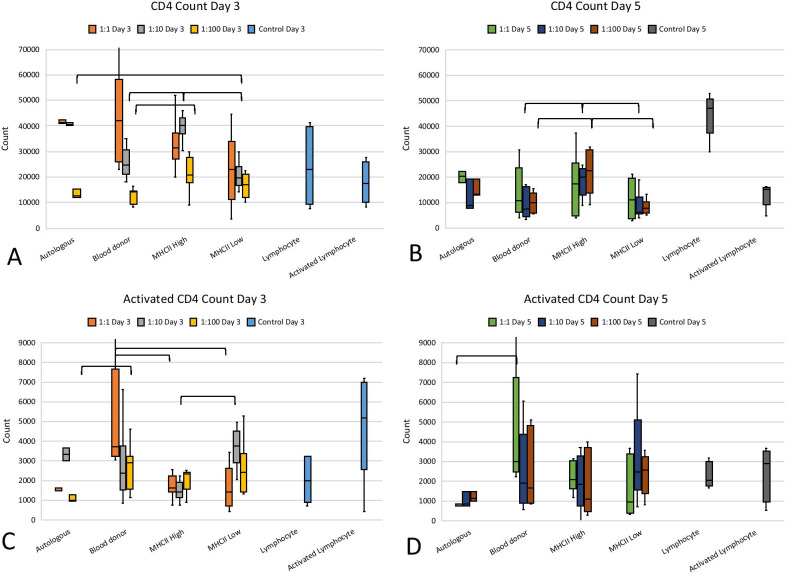
Total CD4 lymphocyte count at Day 3 (**a**) and Day 5 (**b**). Activated CD4 lymphocyte count is shown at Day 3 (**c**) and Day 5 (**d**). Significant differences between the co-culture groups are shown with brackets

Flow cytometric analysis of the PBMCs at Day 3 and Day 5 of co-culture showed a decrease in CD4 populations over time in co-culture, while this count rose in lymphocyte only controls (Fig. 4). There were significantly less CD4 lymphocytes in all of the allogeneic MSC co-cultures at Day 5 as compared to lymphocytes cultured alone (*p* < 0.05).

When the co-culture groups were compared, there were significantly less CD4 lymphocytes in universal blood donor and MHC II-low co-cultures as compared to MHC II-high co-cultures at Day 3 in the 1:10 ratio (*p* = 0.005 and *p* < 0.001, respectively). The decrease in CD4 lymphocytes was also seen at the 1:100 ratio for universal blood donor MSC co-cultures as compared to MHC II-high (*p* = 0.013). Less CD4 lymphocytes were seen in MHC II-low co-cultures as compared to autologous cultures at Day 3 at ratio 1:10 (*p* < 0.001). Similarly, there were significantly less CD4 lymphocytes in blood donor and MHC II-low co-cultures as compared to MHC II-high co-cultures at Day 5 in the 1:10 ratio and 1:100 ratios (*p* = 0.034 and *p* = 0.037 at 1:10 and *p* = 0.035 and *p* = 0.005 at 1:100, respectively).

Activation of CD4 lymphocytes as seen by expression of CD25 was similar for most groups of allogeneic MSCs, the autologous co-cultures, and for lymphocytes cultured alone. This was true except for the universal blood donor co-cultures which contained significantly more activated CD4 lymphocytes than MHC II-high, MHC II-low (ratio 1:1 on Day 3, *p* = 0.021 and *p* = 0.006, respectively), and autologous MSC co-cultures (Day 3 at 1:100, *p* = 0.034; Day 5 at 1:1, *p* = 0.028) (Fig. 4).

### CD8 lymphocyte counts in co-culture decreased over time

**Fig. 5:**
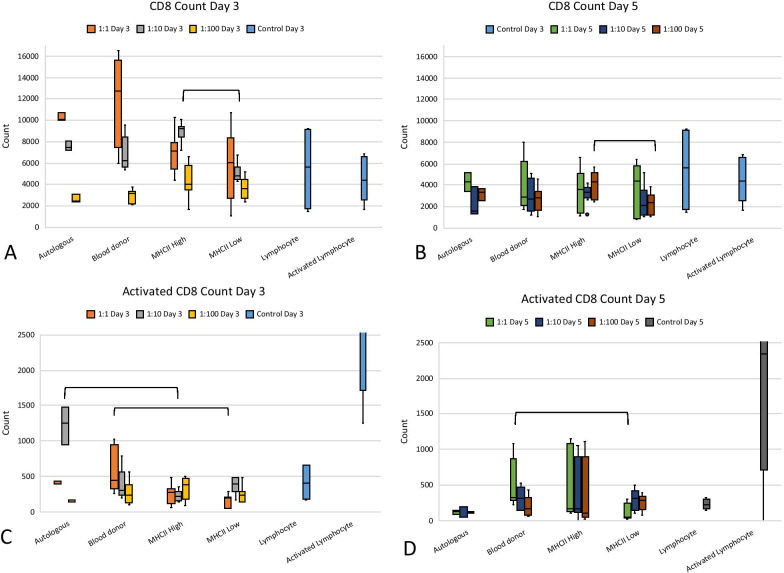
Total count of CD8+ lymphocytes at Day 3 (**a**) and Day 5 (**b**) of co-culture. Activated CD8+ lymphocytes in co-culture with MSCs are shown at Day 3 (**c**) and Day 5 (**d**). Data for co-cultures, activated lymphocytes, and lymphocytes alone are shown at Days 3 and 5. Significant differences between the co-culture groups o are shown with brackets

Median CD8 lymphocyte counts decreased from Day 3 to 5 when co-cultured with autologous or allogeneic MSCs (Fig. 5). At Day 3, co-culture with MHC II-low MSCs led to a significantly lower number of CD8 lymphocytes as compared to MHC II-high co-culture groups at ratios 1:10 and 1:100 (*p* < 0.001 and *p* = 0.045, respectively).

Activation of CD8 lymphocytes as shown by expression of CD25 was generally low and similar to lymphocytes cultured alone. Some significant differences were seen between the groups as shown in Fig. 5, though none of these differences were consistent from one time point to the next nor across more than one ratio of MSC/PBMC.

### B cells in co-culture were consistent over the culture period

**Fig. 6:**
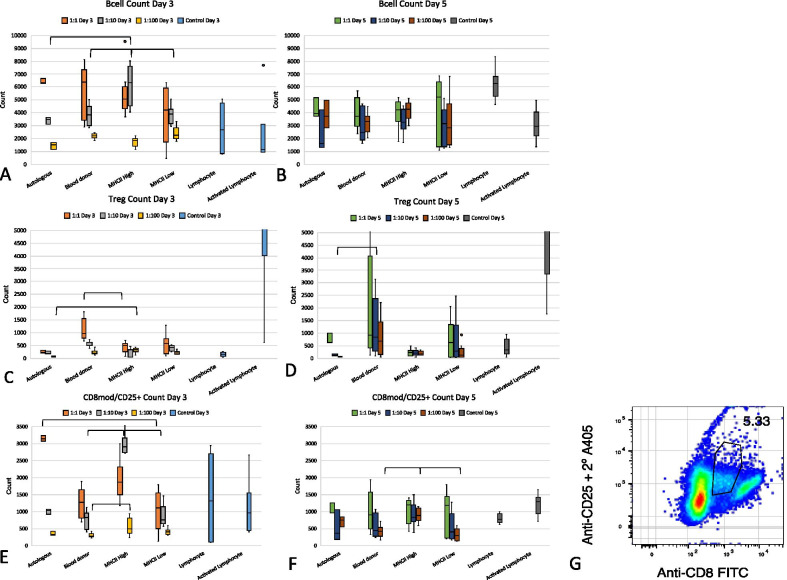
B cell, T cell and CD8moderate/ CD25+ lymphocyte counts in co-culture at Day 3 (**a**, **c**, **e**) and Day 5 (**b**, **d**, **f**). B cell counts increased in PBMC only wells over time. Lower ratios of MSCs to PBMCs caused greater B cell counts. Significant differences between the co-culture groups are shown with brackets. Tregs were increased in 1:1 MSC/PBMC co-cultures as compared to cultures with a greater ratio of PBMCs. Blood donor co-cultures had consistently higher Tregs than other co-cultures. Total CD8moderate/CD25+ lymphocyte count illustrates elevated counts in MHC II-high co-cultures at Day 3 (**e**) and 5 (**f**). A representative sample of CD8moderate/ CD25+ lymphocytes is shown as the gated sample (black circle) (**g**). Significant differences between the co-culture groups are shown with brackets

Flow cytometric evaluation of B cell populations, as shown by CD21 marker expression, showed that the total number of B lymphocytes remained relatively constant between Day 3 and Day 5 in co-culture with MSCs, while control B cells (cultured without MSCs) showed an increase from Day 3 to Day 5 (Fig. 6). Activation of the lymphocytes with PWM did not lead to B cell proliferation (Fig. 6). There were a greater number of B cells when co-culture ratios were low as the 1:1 ratio across all co-cultures had a significantly greater number of B cells than the 1:10 ratio at Day 3 and Day 5 (*p* < 0.001) and a significantly greater number of B cells than the 1:100 ratio at Day 3 (*p* < 0.001).

MHC class-high co-cultures had significantly greater B cell counts at Day 3 (ratio 1:10 than all other co-culture groups; *p* = 0.029, *p* = 0.012, *p* = 0.026, for autologous, universal blood donor, and MHC II-low, respectively). At Day 3 ratio 1:100, MHC II-low co-cultures caused greater B cell count than autologous and MHC II-high co-cultures (*p* = 0.032 and *p* = 0.020, respectively). There were no significant differences between co-culture groups at Day 5.

### Treg lymphocytes increase in universal blood donor and MHC class II-low MSC co-culture with PBMCs

Regulatory T lymphocytes (Tregs) were identified using cell surface antibodies for CD4 and CD25 and intracellular antibodies for FOXP3. Tregs were low in PBMC only cultures (median was < 1% of CD4 cells at Day 3 and Day 5 of culture) and high in PWM activated cultures (median > 60% of CD4 cells at Day 3 and Day 5 of culture) (Fig. 6). The lowest ratio of MSCs/lymphocytes across all co-culture groups was consistently associated with a greater number of Tregs as compared to those co-cultures containing fewer MSCs as compared to lymphocytes (*p* < 0.001 for 1:1 to 1:10 and 1:1 to 1:100 at Day 3) (*p* = 0.002 for 1:1 to 1:10 and *p* = 0.013 for 1:1 to 1:100 at Day 5).

Universal blood donor MSCs were associated with a significant increase in the Treg population as compared to other co-cultures (MHC class II-high MSCs at Day 3, ratio 1:10, *p* < 0.001; autologous MSCs at Day 5, ratio 1:100, *p* = 0.03) (Fig. 6). MHC II-high co-cultures caused a significantly greater number of Tregs as compared to autologous MSCs at Day 3, ratio 1:100 (*p* = 0.003).

**Fig. 7:**
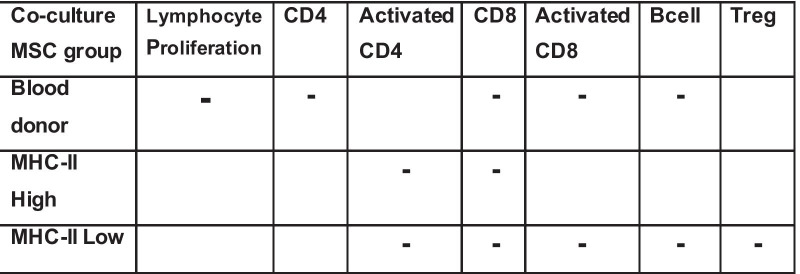
Lymphocyte population dynamics when co-cultured with MSCs. Red arrows show significant increases in inflammatory-type lymphocytes. Green arrows show significant decreases in inflammatory-type lymphocytes or significant increases in anti-inflammatory-type lymphocytes. Black dashes show no significant change as compared to autologous MSC co-cultures

Figure 7 shows a summary of the population dynamics of the CD4, CD8, B cell, and Treg lymphocytes when co-cultured with the MSC groups.

### CD8moderate/CD25+ lymphocytes may be a gamma delta T lymphocytes

A subset of cells not characterizable as CD4 or CD8 T lymphocytes, B cells, or Tregs were apparent upon analysis using antibodies for CD8 and CD25. These cells were moderate in their expression of CD8 and strong in their expression of CD25 and likely fit the description of gamma delta (γδ) T cells (Fig. 6). The absolute number of these cells was high on Day 0 (median across all cultures was 31,900 cells/well) but decreased by Day 3 to less than 3500 across all cultures (Fig. 6). At Day 3 of co-culture, MHC class II-high co-cultures had significantly greater numbers of these possible γδ T lymphocytes as compared to universal blood donor and MHC class II-low co-cultures across more than one MSC/PBMC ratio (Fig. 6). At day 5, MHC class II-high co-cultures had significantly greater numbers of these lymphocytes than universal blood donor and MHC II-low co-cultures at ratio 1:100 (Fig. 6).

### CD4-/CD8-/CD21-/CD25-PBMCs comprise approximately 10% of the population

A final group of PBMCs was consistently identified. These were negative to all of the antibodies used in our panel. These unbound cells may be natural killer cells which are known to lack expression of the antibodies in our panel. There were no significant differences between the co-culture groups at Days 3 and 5 (Additional file 1).

### Neutrophil and MSC co-culture

#### Neutrophil activation was seen in co-cultures with allogeneic MSCs at time point 6 h but had dissipated by time point 12 h

**Fig. 8:**
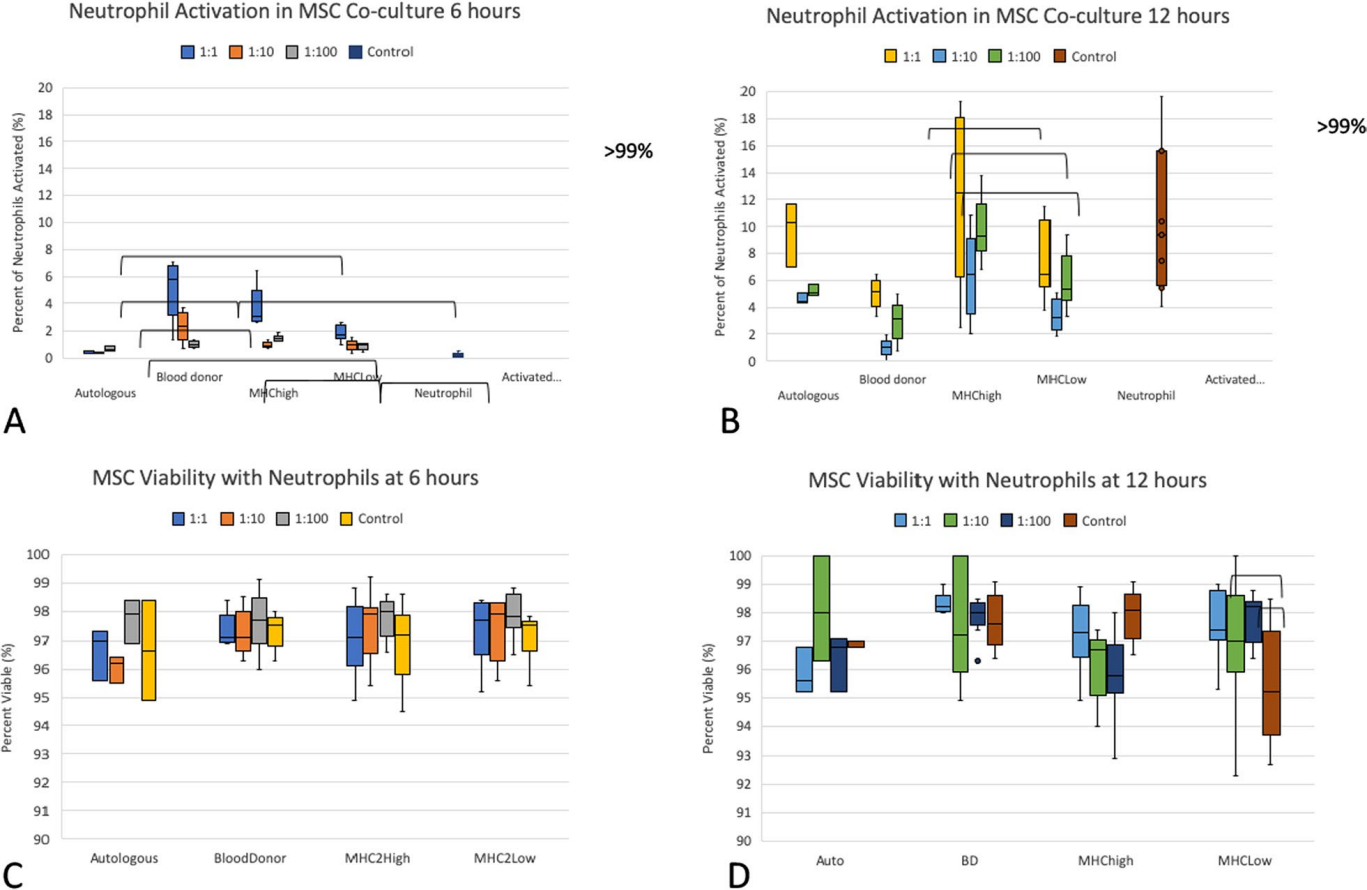
Neutrophil activation and MSC survival in co-culture with neutrophils at time points 6 h (**a**, **c**) and 12 h (**b**, **d**). Ratios of 1 MSC/1 neutrophil, 1MSC/10 neutrophils and 1 MSC/100 neutrophils and control MSCs (no neutrophils) are shown. At time 6 h, universal blood donor and MHC II-high co-cultures had ratios where activation was significantly greater than the autologous co-culture. At time 12 h, only the MCH II-high co-cultures had greater activation as compared to the universal blood donor (BD) co-cultures. Median MSC survival was greater than 95% when co-cultured with neutrophils at time point 6 h (**c**) and 12 h (**d**). The only group with significant viability loss with neutrophils as compared to MSCs cultured alone was the MHC II-high group at 12 h

Some allogeneic MSC groups caused significant activation of neutrophils as compared to autologous MSC co-cultures at 6 h of co-culture (Fig. 8a). Significant differences are shown in Fig. 8 and Table 4. At the 6 h time point, median levels of activation were low for all groups with a median percent of neutrophils that were activated at less than 6%. Both the universal blood donor and the MHC II-high group showed significant increases in neutrophil activation over the other groups at one of the MSC/Neutrophil ratios (Fig. 8 and Table 4). At the 12-h co-culture time point, median neutrophil activation levels increased for most co-cultures and for the neutrophils cultured alone. MHC II-high co-cultures showed increased activation as compared to the universal blood donor co-cultures (Fig. 8 and Table 4).

**Table 4:** Neutrophil activation in co-culture with MSCs

	6 h				12 h			
	1:1	1:10	1:100	Control	1:1	1:10	1:100	Control
Autologous	0.53 (0.09)^a^	0.50 (0.05)^a^	0.66 (0.17)^a^	–	10.3 (2.4)^a,b^	4.5 (0.4)^a,b^	5.1 (0.4)^a,b^	–
Blood donor	5.76 (3.17)^b^	2.40 (1.75)^b^	1.00 (0.34)^a^	–	5.1 (1.5)^a^	1.0 (0.6)^a^	3.2 (2.4)^a^	–
MHC II-high	3.04 (0.08)^b,c^	0.92 (0.30)^a,b^	1.42 (0.31)^b^	–	12.5 (12.9)^b^	6.4 (6.0)^b^	9.3 (2.3)^b^	–
MHC II-low	1.78 (0.82)^a,c^	0.99 (0.41)^a,b^	0.99 (0.37)^a^	–	6.4 (3.7)^a,b^	3.2 (1.7)^a,b^	5.4 (2.8)^a,b^	–
PBMC	–	–	–	0.21 (0.20)	–	–	–	9.3 (9.8)
Activated PBMC	–	–	–	99.9 (0.1)	–	–	–	99.5 (1.6)

Median percent of neutrophils activated and the IQR is shown for co-cultures with the ratio of MSCs/neutrophils and for neutrophils cultured alone. Values within each column which have different letters are significantly different (*p* < 0.05). Autologous, autologous MSC co-culture (*n* = 1); blood donor, universal blood donor MSC co-culture (*n* = 3); MHC II high, MHC II-high MSC co-culture (*n* = 3); MHC II Low, MHC II-low MSC co-culture (*n* = 3); neutrophils, neutrophils alone; activated neutrophils, neutrophils activated with 2.5 µM phorbol myristate acetate (PMA)

### MSC survival had a median of > 95% when co-cultured with neutrophils

MSC survival was assessed using flow cytometry at time 6 h and 12 h of co-culture with neutrophils (Fig. 8). At time point 6 h, no MSC co-cultures had significant loss of viability as compared to those MSCs cultured without neutrophils. At 12 h, only the MHC class II-high groups had a significant loss of viability compared to cultures without neutrophils. The median (IQR) for 1:1 ratio, 1:100 ratio, and MSCs cultured without neutrophils was 96.7% (1.1), 95.1% (0.5), and 98.1% (0.8), respectively.

### Complement-mediated effects on MSCs

#### Complement-mediated MSC death was minimal for both the autologous and allogeneic MSCs

Complement had little effect on MSC viability after one hour in co-culture. Only the MHC II-high MSCs had a significant loss of viability when cultured with complement as compared to those MSCs cultured without active complement (Additional file 1). The median (IQR) survival for MHC II-high MSCs cultured with complement and the MHC II-high MSCs cultured with inactivated complement was 88.3 (3.3) and 90.5 (2.2), respectively (Additional file 1).

### Gene expression in MSC and PBMC co-culture

#### Higher anabolic and anti-inflammatory gene expression is seen in MHC class II-low and universal blood donor MSCs when co-cultured with PBMCs

Gene expression for 10 anti-inflammatory or anabolic genes was measured at Days 3 and 5. MHC class II-low and universal blood donor MSCs were consistently higher in their gene expression as compared to autologous and MHC class II-high MSCs. MHC class II-low MSCs had significantly greater gene expression than autologous and MHC II-high MSCs for the genes encoding CD59, FGF-2, HGF, IDO, IL-10, IL-RA, IL-2, SOX2, and TGF-β1. Universal blood donor MSCs had significantly greater gene expression than autologous and MHC II-high MSCs for the genes encoding FGF-2, HGF, IDO, IL-10, IL-RA, SOX2, and TGF-β1 (Table 5).

**Table 5:** Anabolic gene expression in MSCs co-cultured with PBMCs

		Autologous	Blood donor	MHC II-high	MHC II-low
CD59 Day 3	1:1	3.53 (0.69)^a^	4.24 (3.08)^a^	5.42 (2.69)^a,b^	14.5 (10.4)^b^
	Control	3.68 (0.98)	4.55 (4.52)	7.75 (7.03)	2.31 (1.08)
CD59 Day 5	1:1	9.54 (27.5)	11.5 (10.1)	5.18 (7.53)	9.08 (4.61)
	Control	4.41 (0.77)	2.60 (1.90)	4.74 (2.69)	4.01 (2.08)
FGF-2 Day 3	1:1	852 (21.8)^a,c^	5910 (5300)^b^	450 (112)^a^	2470(2150)^b,c^
	Control	919 (192)	5100 (3690)	955 (1110)	4150 (5550)
FGF-2 Day 5	1:1	1450 (307)^a^	1390 (1260)^a^	473 (397)^b^	1100 (451)^a,c^
	Control	1100 (1040)	3570 (3590)	1820 (1520)	2850 (6170)
HGF Day 3	1:1	41.2 (3.55)^a^	307 (137)^b^	76.4 (64.4)^c^	386 (184)^b^
	Control	166 (43.4)	263 (391)	136 (48.9)	572 (440)
HGF Day 5	1:1	85.8 (17.5)	132 (55.0)	113 (52.3)	119 (14.9)
	Control	189 (80.5)	296 (236)	356 (172)	1050 (519)
IDO Day 3	1:1	123 (26.8)^a^	3850 (1220)^b^	207 (154)^a^	3510 (2170)^b^
	Control	3.90 (1.41)	8.01 (5.27)	45.7 (51.1)	9.90 (11.3)
IDO Day 5	1:1	485 (222)^a^	1420 (1100)^b^	205 (225)^a^	2050 (1900)^b^
	Control	2.16 (1.42)	4.55 (8.86)	5.08 (4.62)	3.29 (2.22)
IL-10 Day 3	1:1	17.0 (1.45)^a^	57.1 (88.4)^b^	32.0 (7.3)^a^	66.4 (45.5)^b^
	Control	1.71 (1.45)	4.32 (1.55)	16.5 (12.9)	24.7 (22.1)
IL-10 Day 5	1:1	29.7 (13.5)	258 (214)	62.4 (17.6)	207 (230)
	Control	5.83 (2.50)	6.71 (3.10)	5.74 (4.76)	7.15 (4.08)
IL-ra Day 3	1:1	27.0 (3.0)^a,b^	76.0 (353)^b,c^	24.4 (14.5)^a^	725 (500)^c^
	Control	2.0 (6.5)	5.7 (3.9)	14.9 (39.3)	18.5 (21.2)
IL-ra Day 5	1:1	78.4 (33.6)^a,b^	65.6 (39.2)^a,b^	29.6 (14.6)^a^	106 (53.9)^b^
	Control	3.2 (2.1)	6.4 (2.3)	5.1 (7.9)	3.2 (2.6)
IL-2 Day 3	1:1	4.7 (0.4)^a^	25.5 (12.3)^a,b^	10.7 (15.9)^a^	38.4 (11.6)^b^
	Control	2.3 (0.5)	3.3 (1.1)	7.8 (1.6)	5.9 (1.3)
IL-2 Day 5	1:1	4.6 (5.2)	11.3 (9.2)	18.5 (4.7)	11.9 (5.6)
	Control	2.2 (0.7)	2.5 (3.0)	1.9 (1.4)	6.0 (4.5)
Sox2 Day 3	1:1	1.0 (0.1)^a,c^	3.6 (2.9)^b^	1.0 (0)^c^	2.5 (2.7)^a,b^
	Control	1.0 (0.2)	1.2 (0.3)	3.7 (10.6)	1.1 (0.2)
Sox2 Day 5	1:1	5.7 (9.7)^a,b^	1.0 (0.6)^a,b^	1.9 (2.4)^a^	1.0 (0)^b^
	Control	1.0 (0)	1.0 (0)	1.0 (0)	1.0 (0)
TGFB1 Day 3	1:1	299 (20)^a^	625 (86)^b^	334 (89)^a^	556 (294)^b^
	Control	422 (71)	255 (54)	283 (112)	249 (46)
TGFB1 Day 5	1:1	411 (101)	601 (320)	460 (213)	669 (384)
	Control	575 (38)	428 (47)	573 (174)	409 (36)
VEGF Day 3	1:1	19,900 (500)	20,700 (19,700)	26,100 (6600)	21,600 (4460)
	Control	2880 (550)	6030 (3380)	3200 (4900)	8170 (7780)
VEGF Day 5	1:1	18,200 (4600)	18,100 (14,600)	23,000 (6800)	14,900 (3570)
	Control	4130 (406)	4410 (1650)	5520 (780)	3930 (5490)

Median RNA copy number and IQR is shown for co-cultures with PBMCs and for MSCs cultured alone (control) at Days 3 and 5 of co-culture. Values for co-cultured MSC gene expression (within each row) which are significantly different are marked with different letters (*p* < 0.05). Autologous, autologous MSC co-culture (*n* = 1); blood donor, universal blood donor MSC co-culture (*n* = 3); MHC II high, MHC II-high MSC co-culture (*n* = 3); MHC II low, MHC II-low MSC co-culture (*n* = 3)

#### Inflammatory gene expression was generally higher for universal blood donor and MHC class II-low MSCs when co-cultured with PBMCs

Gene expression for ten inflammatory or catabolic genes was assessed at Days 3 and 5 of co-culture. Autologous cells were consistently lower in inflammatory gene expression except for ADAMSTS-5 and MMP-13 at Day 5 (Table 6). MHC class II-high MSCs were generally low in expression of these genes except for ADAMSTS-4 at Day 5. Universal blood donor MSC gene expression was significantly higher than the autologous MSCs for its expression of ADAMSTS-4, ADAMSTS-5, CCL2, CXCLB/IL-8, IFNγ, IL-1β, and TNFα. MHC class II-low gene expression was significantly higher than other MSC groups for its expression of ADAMSTS-4, CCL2, CXCLB/IL-8, IFNγ, IL-1b, and TNFα (Table 6).

**Table 6:** Catabolic gene expression in MSCs co-cultured with PBMCs

		Autologous	Blood donor	MHC II-high	MHC II-low
ADAMTS-4 Day 3	1:1	179 (13)^a^	924 (997)^b,c^	371 (137)^a,c^	526 (282)^c^
	Control	66 (30)	28 (20)	90 (44)	34 (8)
ADAMTS-4 Day 5	1:1	253 (50)^a^	882 (444)^b,c^	1087 (805)^b^	671 (606)^a,c^
	Control	108 (27)	85 (21)	117 (183)	50 (25)
ADAMTS-5 Day 3	1:1	2050 (220)^a^	5110 (3940)^b^	2420 (610)^a^	4000 (2200)^a,b^
	Control	1150 (150)	2200 (780)	1390 (1240)	1260 (910)
ADAMTS- Day 5	1:1	6990 (1450)^a^	2490 (3840)^a,b^	2950 (2040)^a^	1540 (110)^b^
	Control	1210 (260)	2230 (680)	1680 (1270)	835 (1170)
CCL2 Day 3	1:1	14,200 (1300)^a^	59,900 (25,400)^b^	15,300 (8800)^a^	76,600 (60,000)^b^
	Control	2110 (330)	4080 (4230)	2840 (1140)	4110 (3470)
CCL2 Day 5	1:1	10,100 (1700)^a,c^	38,200 (11,400)^b^	3440 (4060)^a^	36,900 (19,300)^a,c^
	Control	1820 (100)	2880 (2230)	2190 (830)	2760 (1860)
IL-8 Day 3	1:1	860 (66)^a^	6570 (22,200)^b,c^	2770 (1460)^a,c^	12,800 (17,800)^b,c^
	Control	10.9 (5.4)	146 (234)	9.88 (10.0)	32.2 (30.7)
IL-8 Day 5	1:1	66.2 (95.3)^a^	2370 (3930)^b^	176 (472)^a^	3140 (8670)^b^
	Control	2.64 (1.38)	97.7 (70.1)	9.6 (6.5)	6.31 (12.7)
IFNÝ Day 3	1:1	9.77 (3.88)^a^	392 (164)^b^	6.29 (10.8)^a^	492 (359)^b^
	Control	2.20 (0.53)	5.66 (6.84)	20.8 (28.2)	3.22 (1.27)
IFNÝ Day 5	1:1	21.5 (21.0)^a,c^	546 (618)^b^	13.4 (10.5)^a^	640 (680)^a,c^
	Control	2.41 (3.20)	2.78 (2.59)	2.25 (1.35)	3.93 (3.53)
IL-1b Day 3	1:1	12.4 (6.3)^a^	255 (1050)^b,c^	47.1 (42.2)^a,b^	460 (1457)^c^
	Control	3.95 (1.53)	11.9 (3.5)	5.3 (11.4)	12.6 (9.8)
IL-1b Day 5	1:1	32.1 (23.0)^a^	230 (279)^b^	22.4 (11.7)^a^	352 (1016)^b^
	Control	11.8 (5.0)	13.8 (4.4)	8.15 (9.35)	6.15 (4.16)
IL-6 Day 3	1:1	16,200 (800)^a,b^	50,200(24,400)^a^	6240 (3310)^b^	47,500 (56,200)^b^
	Control	48.9 (14.6)	1360 (1100)	40.5 (52.2)	234 (460)
IL-6 Day 5	1:1	4750 (1520)^a,b^	19,500 (9200)^a^	1010 (2090)^b^	13,400 (6000)^a^
	Control	33.6 (31.8)	541 (111)	24.1 (17.0	79.1 (283)
MMP-13 Day 3	1:1	315 (16)^a,b^	92.7 (6945)^a^	855 (372)^b^	140 (47)^a^
	Control	18.1 (6.9)	181 (195)	1010 (8550)	368 (352)
MMP-13 Day 5	1:1	687 (181)^a^	136 (872)^a^	935 (607)^a^	62.8 (70.8)^b^
	Control	90.3 (19.2)	242 (225)	2190 (14,500)	1010 (1040)
COX2 Day 3	1:1	9400 (810)^a,b^	16,400 (7900)^a^	7610 (2820)^b^	9440 (18,700)^a^
	Control	71.5 (10.3)	3560 (4100)	344 (1350)	1690 (1520)
COX2 Day 5	1:1	8930 (4150)	10,300 (8900)	6290 (26,700)	4990 (1850)
	Control	186 (1220)	6830 (6520)	558 (1280)	612 (1690)
TNFa Day 3	1:1	8.29 (3.65)^a^	48.9 (17.5)^b^	15.1 (8.5)^a^	51.8 (42.1)^b^
	Control	2.17 (1.27	6.22 (1.97)	15.2 (10.8)	14.2 (3.0)
TNFa Day 5	1:1	29.8 (31.9)	52.1 (44.3)	12.2 (23.9)	63.3 (35.5)
	Control	1.77 (0.71)	3.65 (0.99)	5.45 (2.32)	4.61 (1.19)

Median RNA copy number and IQR is shown for co-cultures with PBMCs and for MSCs cultured alone (control) at Days 3 and 5 of co-culture. Values for co-cultured MSC gene expression (within each row) which are significantly different are marked with different letters (*p* < 0.05). Autologous, Autologous MSC co-culture (*n* = 1); blood donor, universal blood donor MSC co-culture (*n* = 3); MHC II high, MHC II-high MSC co-culture (*n* = 3); MHC II Low, MHC II-low MSC co-culture (*n* = 3)

### PBMC and MSC separation

Gene expression was also analyzed on the PBMCs that were co-cultured with the MSC groups. When comparing MSC and PBMC expression in each group, gene expression between these two types of cells varied (Supplementary data). This illustrates that our cell separation method was adequate to remove the PBMCs from the MSCs in each well.

#### Inflammatory gene expression of PBMCs was increased when co-cultured with universal blood donor or MHC class II-high MSCs

Gene expression for the same ten inflammatory genes was analyzed on the PBMCs in co-culture. PBMCs co-cultured with universal blood donor MSCs showed significantly greater expression of the inflammatory genes ADAMSTS-4, ADAMTS-5, CXCL8/IL-8, IL-6, and PTGS2/COX-2 as compared to those cultured with autologous MSCs (Additional file 1). PBMCs co-cultured with MHC class II-low MSCs showed significantly greater expression of the inflammatory genes ADAMTS-5 and CXCL8/IL-8 as compared to those cultured with autologous MSCs (Additional file 1). PBMCs co-cultured with MHC II-low MSCs had significantly lower MMP-13 expression as compared to those cultured with autologous MSCs.

## Discussion

This study was a first of its kind in equine medicine to monitor multiple types of leukocytes in their interaction with MSCs without the presence of external activators. The lack of activation of the leukocytes would allow the immune cells to respond to the MSCs without other contributing factors of inflammation. Although this method may not appropriately represent injured tissues which contain inflammation, our research focused on the basic interaction of the MSCs and leukocytes.

The approach was to use a single leukocyte population to determine variation in reactions across ten different MSC populations. Although the use of one leukocyte population is intrinsically limiting on universality of this data, the number of MSC types and great breadth of assays completed provides us a broad understanding of the interactions occurring between the leukocytes and the MSCs.

Haplotyping revealed that the leukocytes utilized from the Connemara horse were ELA mismatched from all of the allogenic MSCs used in co-culture. Therefore, each of the allogeneic MSCs was equally susceptible to an immune response by the recipient leukocytes, and none of the MSC groups had haplotype matching which may make them less recognizable to the leukocytes in co-culture. It has been hypothesized that the use of haplotype-matched donor MSCs may be the future of allogeneic regenerative medicine when repeat therapy is necessary [56]. As there are greater than 300 ELA subtypes identified [53], finding a matched donor-recipient pair may prove difficult. For this reason, we sought to identify minimally immunogenic MSCs that may be used as a one-time therapy or potentially be utilized repeatedly by rotating the haplotype of the donor MSC. Rotation of allogeneic MSC haplotypes may prevent antibodies being present in the recipient at the time of administration.

The PBMC proliferation rates and cell surface antigens evaluated in this study illustrate that there were no signs of severe negative reactions of PBMCs when cultured with allogeneic MSC. Activation rates of CD4 and CD8 lymphocytes were consistent with those co-cultured with autologous cells (Figs. 4 and 5). The only exception to this was the universal blood donor MSC group which caused greater activation of CD4 lymphocytes at some concentrations.

B lymphocyte numbers were not consistently increased in the face of allogeneic MSCs (Fig. 6). B lymphocyte numbers increased over time when PBMCs were cultured alone, but this was not seen in co-cultures. Antibody production is a common concern for successive allogeneic MSC treatments [48], and it has previously been reported that B cells create antibodies against ELA mismatched allogeneic MSCs which leads to MSC destruction [7, 8]. It is interesting that in our study B cells were not stimulated leading to proliferation when faced with allogeneic MSCs. This may be an indication of B cell suppression by MSCs that will help to provide MSCs with some degree of persistent alloimmunity. There is widespread evidence that human MSCs can also suppress activated B cell responses [6, 15, 23, 25].

MSC-mediated immunosuppression is caused in large part due to an increase in regulatory T lymphocytes which serve to dampen the adaptive immune response and can prevent rejection of foreign cells by the host [63]. In our study, Tregs cells were consistently increased in co-cultures at low MSC/PBMC ratios where MSCs would potentially have the greatest interaction with lymphocytes (Fig. 6). Blood donor MSCs caused a significant increase in Tregs as compared to other MSC groups at both Days 3 and 5, and MHC II-low MSCs showed a somewhat lesser increase though this was not significant (Fig. 6). The increase in Tregs when cultured with MSCs is consistent with previously published human studies [21, 22, 27].

In addition to the already mentioned populations of PBMCs, there were 2 other distinct groups identified on flow cytometry. A CD8moderate/CD25+/CD4- population may represent gamma delta (γδ) T cells, as these cells are known to be negative for CD4, but can be variable in their CD8 expression [1] (Fig. 6). No previous flow cytometry studies have been published on this type of cell in the horse and further assessment of the significance of the loss of these cells in equine co-cultures is required. A subpopulation of CD4-/CD8-/CD21-/CD25-cells was identified (Additional file 1). This population represents approximately 10% of PBMCs and may constitute a population of NK cells based on its lack of marker expression and approximate percent contribution of cells to the PBMC population [42]. Further research and an expanded number of antibodies are needed for appropriate identification.

Neutrophils are often the first line of defense against foreign antigens [33, 40] and therefore would potentially be an initial impediment against the use of allogeneic MSCs. Many studies have shown no increase in activation of neutrophils in the presence of allogeneic MSCs and have shown instead that allogeneic MSCs serve to decrease oxidation and preserve neutrophil viability [43, 51]. Some concern exists when MSCs are used intra-articularly in that a neutrophil influx occurs following MSC administration [4, 14]. In our study, neutrophils were activated upon interaction with allogeneic MSCs, but this activation was minimal and short-lived for the MHC II-low MSC co-cultures. Neutrophil interaction with MSCs showed that the universal blood donor and the MHC II-high allogeneic MSCs at the 6 h time point in co-culture caused greater neutrophil activation than other co-cultures. MHC II-high MSCs consistently caused the highest levels of neutrophil activation (median of all ratios 1.43% at 6 h, 8.9% at 12 h, Fig. 8). This did not have an effect on MSC survival except at high ratios of MHC II-high MSCs to neutrophils at 12 h of co-culture which had a median decreased survival of < 3% (median 95.8% (0.5)) as compared to MHC II-high MSCs cultured alone (median 98.1% (0.8), Fig. 8). In vivo work in the horse found that MHC II-high MSCs caused no greater neutrophil infiltration after an intra-articular injection of allogeneic MSCs as compared to autologous or MHC II-low MSCs [34]. Another study found no difference in neutrophil infiltration in joints treated with autologous MSCs as compared to allogeneic MSCs [4]. It is possible that the significantly increased neutrophil activation seen in the allogeneic co-cultures is so mild and transient that the activation of neutrophils is not clinically significant.

Complement has been considered to be another impediment to allogeneic MSC use as complement can flag the foreign material for phagocytosis or cause cell lysis by forming a membrane attack complex [44]. One group studied human MSC survival in the presence of complement and found > 40% of the MSCs were damaged upon incubation with complement [38, 39]. In contrast, we found complement-mediated cytotoxicity did not cause consistent MSC death in cultures with active complement as compared to inactivated complement. Even in the only MSC culture that showed any significant loss of MSC viability, the MHC II-high MSC culture, this loss of viability was only 2.2% of MSCs (Additional file 1).

Gene regulation of allogeneic MSCs in co-culture is highly variable and appears to relate to the level of MHC II expression of the MSCs. MSCs are known to deliver anabolic factors such as TGF-β1, FGF, and G-CSF; anti-inflammatory factors such as IL-1RA and IDO1; and immunomodulating factors such as CXCLB/IL8 and IFN- γ [3, 11, 20]. Two groups of MSCs, the blood donor and the MSC II-low groups, increased their gene expression of these anabolic genes.

Several of the genes expressed in greater amounts in the blood donor and MHC II-low groups were genes aimed at suppressing the immune system. Indoleamine-pyrrole 2,3-dioxygenase (IDO1) quells T lymphocyte responses and leads to immune tolerance whose effect alone can determine the difference between organ rejection and acceptance [24]. IL-2 binding directly to T lymphocytes causes upregulation of regulatory T cells (Tregs) and increases activation-induced cell death for lymphocytes. Regulatory T cells, cells crucial to the immunosuppressive ability of MSCs, were consistently elevated in lymphocyte co-cultures with universal blood donor MSCs (Fig. 6). Interestingly, this group had a higher level of anabolic and catabolic gene expression, including TGFβ and IFNγ. This is line with the findings of [64] who found that pretreatment of MSCs with TGFβ and IFNγ resulted in MSCs that had a greater capability of forming Tregs. MSCs appear to need some sort of activation of their own to assist in their ability to implement their immunosuppressive effects [37].

Catabolic molecules such as TNF- α, IL1β, aggrecanases, and MMP-13 are commonly upregulated in the face of inflammation [36, 46]. In our gene expression assay, several catabolic factors were increased in blood donor and the MSC II-low co-cultures. This seems to contradict our other data showing decreased inflammation and leukocyte activation when leukocytes were co-cultured with universal blood donor and the MSC II-low MSCs.

The expression of IFNγ in our universal blood donor and MHC II-low co-cultures is especially interesting for two reasons. First, IFNγ can cause increased expression of immunosuppressive genes such as IDO1, HGF, and PGE2/COX2 [37, 49]. In our cultures where IFNγ was increased, these genes were significantly upregulated (Tables 5 and 6). A previous equine MSC study has shown inflammatory licensing by pretreatment of MSCs with IFNγ had superior immunosuppressive effects as compared to non-pre-treated MSCs [11]. Secondly, in both the universal blood donor and MHC II-low groups, where IFNγ gene expression was increased in both the MSCs and the lymphocytes, a significant increase in MHC II expression was seen on the surface of the MSCs as compared to those cells cultured alone. This is consistent with previous work which showed the treatment of MSCs with IFNγ caused increased MHC II expression [30].

From the gene expression data, it is clear that the universal blood donor group and the MHC II-low MSCs were more metabolically active in both the anabolic and catabolic gene categories. Researchers must determine whether the more metabolically active MSCs would be more beneficial as a therapy as compared to the less metabolically active groups, the MHC II-high and autologous MSCs. To better achieve this goal, the alterations to the co-cultured leukocyte population must be examined. As previously discussed, there was no significant neutrophil activation and no decrease in complement-mediated viability in the MHC-low and universal blood donor MSC co-cultures. When considering the interaction of our MSCs with lymphocytes in the current study, MHC II-low MSCs showed only beneficial decreases in lymphocyte proliferation and total CD4 lymphocyte count as compared to autologous MSC co-cultures (Figs. 3 and 4). Universal blood donor MSC co-cultures had an elevated activated CD4 lymphocyte count, but this included increased numbers of Tregs which would serve to decrease an immune response. Only, the MHC II-high MSCs repeatedly showed increased lymphocyte activation.

MSCs have been shown to go through a phenotypic and genotypic metamorphosis when they interact with the immune system [11]. For MHC II-low MSCs, this change in structure and expression appears to affect the cells in a manner that may be preferential when used as an allogeneic treatment. The influence of the origin of these cells from universal blood donor horses or non-blood donor horses does not appear to be markedly significant. MHC II-low MSCs prevent proliferation of PBMCs, increase expression of both anabolic and catabolic genes, decrease activation of neutrophils, and maintain viability when exposed to complement. There were minimal differences between autologous and allogeneic MSCs in their effects on the activation and differentiation of lymphocytes. MHC II-high allogeneic MSCs were the only group of allogeneic MSCs that repeatedly showed increased lymphocyte activation. Some inflammatory gene expression increased in MHC II-low co-cultures, but a reciprocal anti-inflammatory gene response was also seen. These MHC II-low MSCs appear to be activated in the recipient environment to perform immunosuppressive and anabolic functions.

## Conclusion

From the results of this body of in vitro work, we conclude that bone marrow-derived, low passage number MHC II-low MSCs from healthy donors have minimal negative effects on an allogeneic leukocyte population in vitro. This includes the lack of lymphocyte and neutrophil activation and a lack of B cell proliferation. Allogeneic MSCs maintained a high level of viability through all testing, and the MHC II-low MSCs upregulated both their inflammatory and catabolic gene profiles in response to lymphocyte co-culture.

## Supplementary Information

**Additional file 1**. **Table S1.** Antibodies used for flow cytometry assays. **Table S2.** Haplotype analysis shows mis-matched ELA. **Table S3.** Accession numbers for genes used in NanoString assays. **Figure S1.** Flow cytometry gating scheme for lymphocytes. **Figure S2.** Flow cytometry gating scheme for MSCs. **Figure S3.** CD4-/CD8-/CD21-/CD25- PBMCs are shown at days 3 and 5 of co-culture with MSCs. **Figure S4.** MSC survival with complement. MSC survival was not significantly different between cells cultured in active or inactivated complement except for the MHC II-high MSC group which showed a 3% decrease in viability with active complement as compared to inactive complement. *Figure S5.* Day 3 catabolic gene expression in MSC and PBMC co-cultures. Mean MSC RNA copy number is listed in blue. Mean PBMC RNA copy number is listed in orange. **Figure S6.** Day 5 catabolic gene expression in MSC and PBMC co-cultures. Median MSC RNA copy number is listed in blue. Median PBMC RNA copy number is listed in orange. **Figure S7.** Day 3 anabolic gene expression in MSC and PBMC co-cultures. Median MSC RNA copy number is listed in blue. Median PBMC RNA copy number is listed in orange. **Figure S8.** Day 5 anabolic gene expression in MSC and PBMC co-cultures. Median MSC RNA copy number is listed in blue. Median PBMC RNA copy number is listed in orange. **Figure S9.** Inflammatory gene expression is shown for PBMCs in culture with MSCs, PBMCs alone, or PBMCs with activation media. Median RNA copy number of inflammatory genes expressed by PBMCs in shown. Cultures of 1 MSC:1 Lymphochyte and control MSCs (no lymphocytes) are shown. PBMCs cultured with universal blood donor MSCs had higher levels of inflammatory gene expression as compared to those cultured with autologous MSCs in 5 of 10 genes examined.

## Data Availability

Data are available at https://dataverse.harvard.edu/dataset.xhtml?persistentId=doi:10.7910/DVN/HMKGJU.

## Abbreviations

CCL: Chemokine ligand
CXCL8: C-X-C motif chemokine ligand 8
CD: Cluster of differentiation
ELA: Equine lymphocyte antigen
FGF: Fibroblast growth factor
HGF: Hepatocyte growth factor
IL: Interleukin
IL-RA: Interleukin receptor antagonist
IFN: Interferon
IDO1: Indoleamine-pyrrole 2,3-dioxygenase
MHC: Major histocompatibility class
MMP: Matrix metalloproteinase
MSC: Mesenchymal stromal cell
PTGS-2/COX-2: Cyclooxygenase-2
Tregs: T regulatory cells
TNF: Tumor necrosis factor
TGF: Thyroid growth factor
VEGF2: Vascular endothelial growth factor 2
