# Supplementary material for: Immune response to allogeneic equine mesenchymal stromal cells
Source: Stem Cell Res Ther. 2021 Nov 12;12:570. doi: 10.1186/s13287-021-02624-y (PMC8588742; doi:10.1186/s13287-021-02624-y)
Supplement: Supplementary file 1 — Additional file 1. Table S1. Antibodies used for flow cytometry assays. Table S2. Haplotype analysis shows mis-matched ELA. Table S3. Accession numbers for genes used in NanoString assays. Figure S1. Flow cytometry gating scheme for lymphocytes. Figure S2. Flow cytometry gating scheme for MSCs. Figure S3. CD4-/CD8-/CD21-/CD25- PBMCs are shown at days 3 and 5 of co-culture with MSCs. Figure S4. MSC survival with complement. MSC survival was not significantly different between cells cultured in active or inactivated complement except for the MHC II-high MSC group which showed a 3% decrease in viability with active complement as compared to inactive complement. Figure S5. Day 3 catabolic gene expression in MSC and PBMC co-cultures. Mean MSC RNA copy number is listed in blue. Mean PBMC RNA copy number is listed in orange. Figure S6. Day 5 catabolic gene expression in MSC and PBMC co-cultures. Median MSC RNA copy number is listed in blue. Median PBMC RNA copy number is listed in orange. Figure S7. Day 3 anabolic gene expression in MSC and PBMC co-cultures. Median MSC RNA copy number is listed in blue. Median PBMC RNA copy number is listed in orange. Figure S8. Day 5 anabolic gene expression in MSC and PBMC co-cultures. Median MSC RNA copy number is listed in blue. Median PBMC RNA copy number is listed in orange. Figure S9. Inflammatory gene expression is shown for PBMCs in culture with MSCs, PBMCs alone, or PBMCs with activation media. Median RNA copy number of inflammatory genes expressed by PBMCs in shown. Cultures of 1 MSC:1 Lymphochyte and control MSCs (no lymphocytes) are shown. PBMCs cultured with universal blood donor MSCs had higher levels of inflammatory gene expression as compared to those cultured with autologous MSCs in 5 of 10 genes examined. [file 13287_2021_2624_MOESM1_ESM.docx]

**Additional file 1**

| **Antibody Clone** | **Distributer, Catalog number** | **Conjugated fluorochrome or secondary antibody** | **Host Species** | **Ig Type** | **Dilution** |
| --- | --- | --- | --- | --- | --- |
| CD4 CVS4 | US Biological, 227417-ML405 | MaxLight650 | Mouse | IgG1 | 1:700 |
| CD8 CVS8 | BioRad, MCA2385F | FITC | Mouse | IgG1 | 1:200 |
| CD21 CA2.1D6 | AbCam, ab34124 | PE | Mouse | IgM | 1:5 |
| CD25/IL-2 R alpha | RND Sytsems, AF-223-NA | Donkey Anti-Goat IgG H&L (Alexa  Fluor® 405) | Goat | IgG | 1:10 |
| FOXP3 FJK-16s | eBioscience, 17-5773-82 | PE-Cyanine7 | Rat | IgG2a | 1:100 |
| MHC class I CVS22 | BioRad, MCA1086 | RPE | Mouse | IgG | 1:10 |
| MHC class II CVS20 | Bio-Rad, MCA1085F | FITC | Mouse | IgG1 | 1:100 |

**Additional file 1: Table S1. Antibodies used for flow cytometry assays.**

**Additional file 1: Table S2. Haplotype analysis shows mis-matched ELA haplotypes of the Connemara (horse 10) and the other animals.**

| Gene | RefSeq Accession number |
| --- | --- |
| TGF-B1 | NM_001081849 |
| IL-1RA | NM_001082525.2 |
| CD-59 | XM_023653832.1 |
| FGF-2 | NM_001195221.1 |
| IDO-1 | XM_014736538.2 |
| IL-10 | NM_001082490.1 |
| VEGF-a | NM_001081821.1 |
| SOX2 | XM_023623361.1 |
| PTGS-2/ COX-2 | NM_001081775.2 |
| IL-1b | XM_001495926.5 |
| IL-2 | NM_001085433.2 |
| TNFa | NM_001081819.2 |
| Hepatocyte GF | XM_014739139.2 |
| IFNgamma | NM_001081949.1 |
| CXCL8/IL-8 | NM_001083951.2 |
| IL-6 | NM_001082496 |
| MMP-13 | NM_001081804.1 |
| ADAMTS-4 | NM_001111299.2 |
| ADAMTS-5 | XM_003364218 |
| CCL2 | NM_001081931.2 |
| TBP | XM_014738168 |
| GUSB | XM_023655543 |
| PPIA | XM_001496943.5 |
| YWHAZ | XM_014728222.2 |

**Additional file 1: Table S3. Accession numbers for genes used in NanoString assays.**


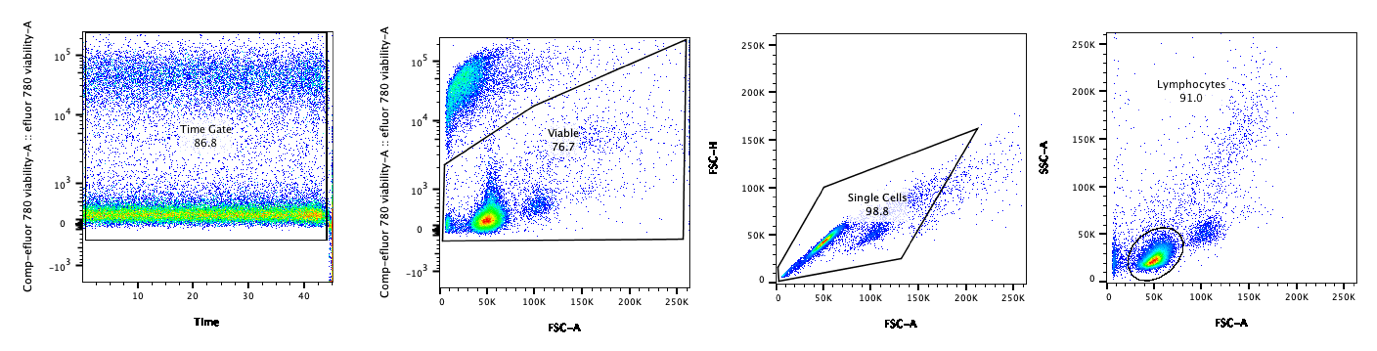


**Additional file 1: Figure S1. Flow cytometry gating scheme for lymphocytes.**


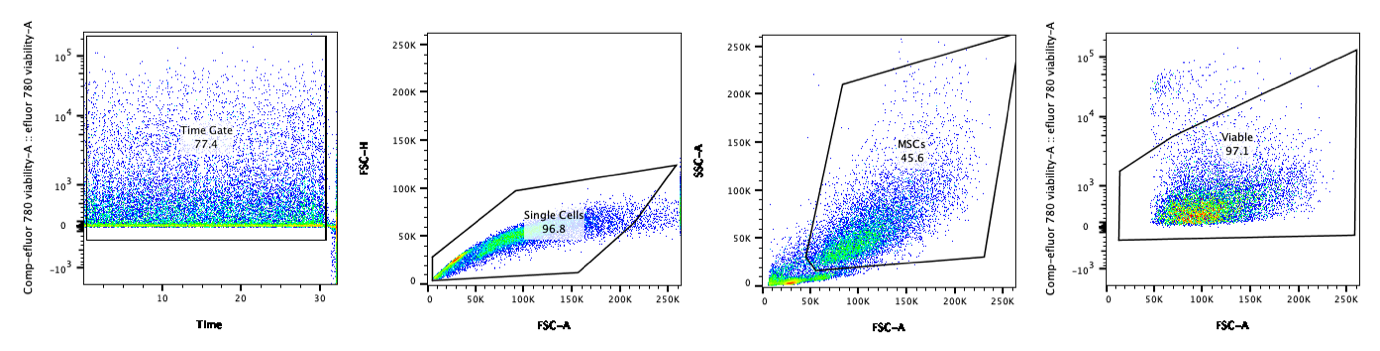


**Additional file 1: Figure S2. Flow cytometry gating scheme for MSCs.**

**Additional file 1: Figure S3. CD4-/CD8-/CD21-/CD25- PBMCs are shown at days 3 and 5 of co-culture with MSCs.**

**Additional file 1: Figure S4. MSC survival with complement.** MSC survival was not significantly different between cells cultured in active or inactivated complement except for the MHC II-high MSC group which showed a 3% decrease in viability with active complement as compared to inactive complement.

**Additional file 1: Figure S5. Day 3 catabolic gene expression in MSC and PBMC co-cultures**. Mean MSC RNA copy number is listed in blue. Mean PBMC RNA copy number is listed in orange.

**Additional file 1: Figure S6. Day 5 catabolic gene expression in MSC and PBMC co-cultures.** Median MSC RNA copy number is listed in blue. Median PBMC RNA copy number is listed in orange.

**Additional file 1: figure S7.** Day 3 anabolic gene expression in MSC and PBMC co-cultures. Median MSC RNA copy number is listed in blue. Median PBMC RNA copy number is listed in orange.

**Additional file 1: Figure S8.** Day 5 anabolic gene expression in MSC and PBMC co-cultures. Median MSC RNA copy number is listed in blue. Median PBMC RNA copy number is listed in orange.


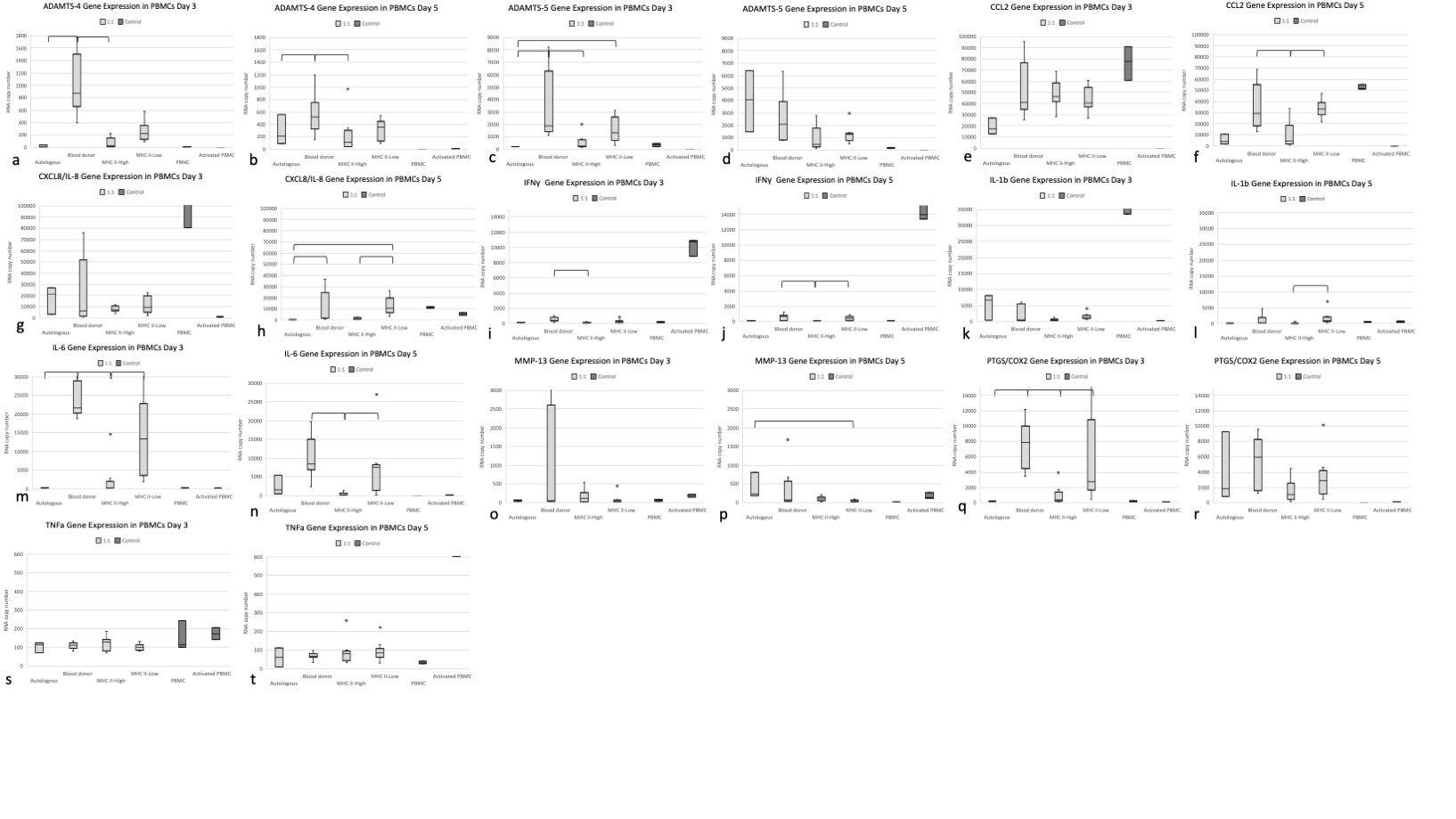


**Additional file 1: Figure S9. Inflammatory gene expression is shown for PBMCs in culture with MSCs, PBMCs alone, or PBMCs with activation media.** Median RNA copy number of inflammatory genes expressed by PBMCs in shown**.** Cultures of 1 MSC:1 Lymphochyte and control MSCs (no lymphocytes) are shown. PBMCs cultured with universal blood donor MSCs had higher levels of inflammatory gene expression as compared to those cultured with autologous MSCs in 5 of 10 genes examined.
